# Supplementary material for: HATRIC-based identification of receptors for orphan ligands
Source: Nat Commun. 2018 Apr 17;9:1519. doi: 10.1038/s41467-018-03936-z (PMC5904110; doi:10.1038/s41467-018-03936-z)
Supplement: Supplementary file 1 — Supplementary Information [file 41467_2018_3936_MOESM1_ESM.docx]

**HATRIC-based identification of receptors for orphan ligands**

Sobotzki *et al.*

Supplementary Note 1: Synthesis of HATRIC

Synthesis of compound **3**

To a solution of **2** (6.59 g, 50.2 mmol, 1.1 equiv) in DMF (150 mL) was added triethylamine (5.08 g, 50.2 mmol, 1.1 equiv), followed by the NHS-ester **1** (15.0 g, 45.7 mmol, 1.0 equiv). The reaction mixture was strirred for 3 h at room temperature. Following aqueous work up the product was purified by re-crystallization from hexanes/ethyl acetate (1:1, 350 ml) to give **3** (17.5 g, 90%) as white crystals.

Synthesis of compound **5**

To a solution of **4** (15.0 g, 56.5 mmol, 1.0 equiv) in DMF (100 mL) was added DMAP (2.76 g, 22.6 mmol, 0.4 equiv), followed by *tert*-butanol (12.6 g, 170 mmol, 3.0 equiv), EDC∙HCl (15.2 g, 79.0 mmol, 1.4 equiv) and Hünig’s base (23.7 mL, 136 mmol, 2.4 equiv). The reaction mixture was stirred for 14 h at room temperature. Following aqueous work up the product was purified by flash column chromatography (hexanes/ethyl acetate 20:1, then 3:1) to give **5** (14.6 g, 81%) as a colorless oil.

Synthesis of compound **6**

To a solution of **5** (14.0 g, 43.6 mmol, 1.0 equiv) in THF/water (5:1, 100 mL) was added acetic acid (2.64 g, 44.0 mmol, 1.0 equiv), followed by Pd/C (1.85 g of 10 wt% loading, 1.74 mmol, 4 mol%). The reaction mixture was stirred at room temperature under an atmosphere of H_2_ for 16 h. Then the reaction mixture was centrifuged, the supernatant was filtered through celite, concentrated under reduced pressure and the resulting oil was dried *in vacuo*. The resulting oil was dissolved in DCM (150 mL) and treated with triethylamine (15.2 mL, 109 mmol, 2.5 equiv) and succinic anhydride (4.79 g, 47.9 mmol, 1.1 equiv) and was stirred at room temperature for 3 h. Following aqueous work up the product was purified by flash column chromatography (DCM, then DCM/MeOH 90:10) to give **6** (11.7 g, 94%) as a white solid.

Synthesis of compound **8**

To a solution of **7**^[1]^ (10.8 g, 30.3 mmol, 1.0 equiv in CH_2_Cl_2_ (400 mL) at 4 °C was added triethylamine (5.29 mL, 37.9 mmol, 1.3 equiv), followed by allyl chloroformate (3.56 mL, 33.4 mmol, 1.1 equiv). After 15 min, the reaction was allowed to warm to room temperature and was stirred for 2 h. Following aqueous work up the product was purified by flash column chromatography (hexanes/ethyl acetate 1:1, then ethyl acetate) to give **8** (11.0 g, 90%) as a colorless oil.

Synthesis of compound **10**

To a solution of **8** (2.83 g, 7.00 mmol, 1.2 equiv) in CH_2_Cl_2_ (10 mL) was added TFA (9 mL) and the reaction mixture was stirred for 30 min at room temperature. Then, toluene (20 mL) was added and the mixture was concentrated under reduced pressure. Co-evaporation with toluene (3 x 30 mL) resulted in a brown oil, which was dried *in vacuo*. The residue was re-dissolved in DMF (20 mL), **9** (2.85 g, 6.09 mmol, 1.0 equiv) was added, followed by Hünig’s base (3.40 mL, 24.4 mmol, 4.0 equiv) and HATU (2.55 g, 6.70 mmol, 1.1 equiv). The reaction mixture was stirred at room temperature for 30 min. Following aqueous work up the product was purified by flash column chromatography (CH_2_Cl_2_, then CH_2_Cl_2_/MeOH 96:4) to give **10** (4.53 g, 99%) as a colorless oil, that solidified upon standing.

Synthesis of compound **11**

To a solution of **10** (6.00 g, 7.95 mmol, 1.0 equiv) in CH_2_Cl_2_ (25 mL) was added TFA (15 mL) and the reaction mixture was stirred at room temperature for 1 h. Then, toluene (30 mL) was added and the mixture was concentrated under reduced pressure. Co-evaporation with toluene (3 x 30 mL) resulted in an oil, which was dried *in vacuo*. The residue was re-dissolved in DMF (20 mL) and **3** (3.01 g, 8.74 mmol, 1.1 equiv) was added, followed by Hünig’s base (6.25 mL, 35.8 mmol, 4.5 equiv) and HATU (3.32 g, 8.74 mmol, 1.1 equiv). The reaction mixture was stirred at room temperature for 30 min. Following aqueous work up the product was purified by flash column chromatography (CH_2_Cl_2_, then CH_2_Cl_2_ /MeOH 9:1) to give **11** (5.90g, 76%) as a colorless oil.

Synthesis of compound **12**

To a solution of **11** (5.85 g, 5.96 mmol, 1.0 equiv) in CH_2_Cl_2_ (12 mL) was added TFA (9 mL) and the reaction mixture was stirred for 1 h at room temperature. Then, toluene (30 mL) was added and the mixture was concentrated under reduced pressure. Co-evaporation with toluene (3 x 30 mL) resulted in an oil which was dried *in vacuo*. The residue was dissolved DMF (25 mL), treated with 6-(2-(*tert*-butoxycarbonyl)hydrazinyl)nicotinic acid^[2]^ (1.79 g, 7.06 mmol, 1.2 equiv), Hünig’s Base (4.69 mL, 26.8 mmol, 4.5 equiv) and HATU (2.49 g, 6.56 mmol, 1.1 quiv) and the reaction mixture was stirred at room temperature for 30 min. Following aqueous work up the product was purified by flash column chromatography (CH_2_Cl_2_, then CH_2_Cl_2_/MeOH 9:1) to give **12** (5.01 g, 75%) as a colorless foamy solid.

Synthesis of compound **13**

To a solution of **12** (4.99 g, 4.47 mmol, 1.0 equiv) in DMF (15 mL) was added piperidine (2 mL) and the reaction mixture was stirred for 1 h at room temperature. Then toluene (15 mL) was added and the mixture was concentrated under reduced pressure. Co-evaporation with toluene (3 x 15 ml) resulted in an oil which was dried *in vacuo*. The residue was dissolved in DMF (15 mL) and treated with **6** (1.54 g, 5.36 mmol, 1.2 equiv), triethylamine (2.65 mL, 19.0 mmol, 4.3 mmol) and HATU (1.87 g, 4.92 mmol, 1.1 equiv) and the reaction mixture was stirred at room temperature for 30 min. Following aqueous work up the product was purified by flash column chromatography (CH_2_Cl_2_ then CH_2_Cl_2_/MeOH 15:1 to 9:1) to give **13** (4.69 g, 90%) as a colorless foamy solid.

Synthesis of compound **14**

To a solution of **13** (274 mg, 0.24 mmol, 1.0 equiv) in MeOH (1.5 mL) was added diethylamine (98 mL, 0.94 mmol, 4.0 equiv), followed by a solution of Pd(PPh_3_)_4_ (14.0 mg, 12.0 mmol, 5 mol%) and PPh_3_ (6.2 mg, 24 mmol, 10 mol%) in CH_2_Cl_2_ (3 mL). After 30 min at room temperature, the reaction mixture was diluted with toluene (10 mL) and concentrated under reduced pressure. Co-evaporation with toluene (2 x 15 mL) resulted in an oil which was dried *in vacuo*. The residue was dissolved in DMF (4.5 mL) and treated with γ-azidobutyric acid^[3]^ (0.040 g, 0.306 mmol, 1.3 equiv), triethylamine (49 mL, 0.35 mmol, 1.5 equiv) and HATU (0.099 g, 0.259 mmol, 1.1 equiv) and the reaction mixture was stirred at room temperature for 1 h. Following evaporaton of solvent the product was purified by flash chromatography (CH_2_Cl_2_, then CH_2_Cl_2_/MeOH 9:1 to 4:1) and then by reversed phase C-18 chromatography (water/acetonitrile 3:1 to 1:1) to give **14** (160 mg, 57%).

Synthesis of compound **15**

An aq. 6 M HCl solution (2 mL) was added to **14** (127 mg, 0.11 mmol, 1.0 equiv) and the solution was stirred at room temperature. After 20 min, the clear solution was cooled in an ice-bath, an aq. 2 M NaOH solution (6.2 mL, previously titrated against HCl solution) was added and the neutralized solution was lyophilized. The residue was suspended in MeOH/acetone (1:1, 10 ml) and the volatiles were evaporated under reduced pressure at 40 °C. This procedure was repeated 2 more times. Then, after suspending in MeOH/acetone (1:1, 10 ml) the suspension was filtered, the solids were washed with MeOH/acetone (1:1, 100 ml) and the filtrate was evaporated. The crude product was purified by reversed-phase column chromatography (H_2_O/acetone/acetonitrile 90:9:0 to 85:10:5) to give the product (61 mg, 52%) of sufficient purity to be used without further purification in the next step.

Synthesis of HATRIC (**16**)

To a suspension of **15** (61 mg, 57 mmol, 1.0 equiv) in DMF (2.5 mL) was added Hünig’s base (20.0 μL, 114 mmol, 2.0 equiv) was added, followed by *N*-hydroxysuccinimide (13.0 mg, 114 mmol, 2.0 equiv) and *N*,*N*'-disuccinimidyl carbonate (29.0 mg, 114 mmol, 2.0 equiv). The reaction mixture was then stirred at 40 °C for 2 h. Following co-evaporation with toluene (3 x 10 mL) the product was purified by size exclusion chromatography using sephadex LH-20 (CH_2_Cl_2_/acetone/MeOH 8:1:1) to give **16** (54 mg, 83%) as an orange solid.

Supplementary Note 2: Synthesis of folic acid derivative^[4]^ and folic acid-HATRIC conjugate

Synthesis of compound **17**

To a solution of Fmoc-Glu-O-t-Bu (2.13 g, 5.00 mmol, 1.25 equiv) in DMF (100 mL) was added **7** (1.28 g, 4.00 mmol, 1.00 quiv), followed by Hünig’s base (0.7 mL, 4.0 mmol, 1.0 quiv) and HATU (1.52 g, 4.00 mmol, 1.00 quiv) and was stirred for 1 h. Following aqueous work up the product was purified by flash chromatography (EtOAc) to give **17** (2.36 g, 81%) as a highly viscous oil

Synthesis of compound **18**

To a solution of **17** (1.65 g, 2.27 mmol, 1.00 quiv) in DMF (4.5 mL) was added piperidine (0.90 mL, 9.1 mmol, 4.0 equiv) and the reaction was stirred at room temperature for 16 h. Following evaporation of solvent the product was purified by flash chromatography (DCM/MeOH) to give **18** (0.83 g, 72%) as a yellowish oil.

Synthesis of compound **19**

To a solution of petroic acid (50 mg, 0.15 mmol, 1.0 quiv) in DMSO (4 mL) was added TBTU (60 mg, 0.19 mmol, 1.3 quiv), followed by HOBT (36 mg, 0.19 mmol, 1.3 quiv) and triethylamine (83 µL, 0.60 mmol, 4.0 quiv) and the reaction was stirred at 50 °C for 90 min. Then a solution of **18** (94 mg, 0.19 mmol, 1.3 equiv) in DMSO (2 mL) was added and the reaction was stirred at 50 °C for 24 h. After precipitation of the crude product with cold ether the product was purified by preparative HPLC to give **19** (63 mg, 53%) as a yellow solid.

**19** (6.8 mg, 8.5 µmol, 2.0 quiv) was dissolved in CHCl_3_/TFA (3:1, 0.2 mL) and stirred at room temperature for 1 h. After evaporation of volatiles the crude product was dissolved in DMSO/DMF (2:1, 0.4 mL) and triethylamine (2.4 µL, ) was added, followed by HATRIC (5.0 mg, 4.3 µmol, 1.0 quiv). The reaction was stirred at room temperature for 16 h. Following removal of solvent the product was purified by size exclusion chromatography using sephadex LH-20 (DCM/acetone/MeOH 8:1:1) to give the folic acid-HATRIC conjugate (4.8 mg, 66%) as a highly viscous oil.

|  |
| --- |
| **Supplementary Figure 1:** **Cytotoxicity of aniline and aniline-derived organocatalysts on MDA-MB 231.** MDA-MB 231 cells (20.000 cells/well in a 96-well plate) were treated with the indicated concentrations of catalyst in DMEM (pH adjusted to 7.4, 1% Pen/Strep) for 1.5h at 37°C. Supernatant was replaced by 100ul DMEM with 10% alamarBlue™ reagent (ThermoScientific) and incubated for 5h at 37°C in the dark. Assay was read out by a fluoreader (Ex: 545nm, Em: 590nm, automatic gain). |

|  |
| --- |
| **Supplementary Figure 2:** **HATRIC co-localizes with cell surface staining as shown by confocal microscopy imaging**. RED: HATRIC-Amine-Cy3, GREEN: Sulfo-NHS-Cy5, BLUE: Hoechst. HATRIC was pre-coupled to equimolar amine-Cy3 (Lumiprobe) in 25mM HEPES (pH 8.2) for 1.5h at RT and 300rpm in the dark. MDA-MB-231 cells cultured on coverslips were oxidized with 1ml 1.5mM sodium periodate in PBS, pH 6.5 for 15min and labeled with 6µM HATRIC-Cy3 or amine-Cy3 (Control w/o HATRIC) in 1ml PBS with 5mM 5-MA (pH 7.4) for 1.5h at 4°C shaking in the dark. As a cell surface marker, cells were labeled with 0.5ml 1mM sulfo-NHS-Cy5 (Lumiprobe). Nuclei were stained with 0.5ml 1µg/ml Hoechst (Molecular probes H1399) for 10min at 4°C. Cells were fixed with 4% paraformaldehyde for 10min at RT, mounted with anti-fade mounting medium (Molecular Probes Prolong Gold Antifade reagent P36934) and analysed by confocal microscopy (Leica TCS SP2). For a permeabilized control, cells were first stained with sulfo-NHS-Cy5 and fixed, and then permeabilized with 0.1% Triton X-100 for 10min at RT, before oxidation and labeling with HATRIC-Cy3. |

|  |
| --- |

**Supplementary Figure 3: Volcano plot from EGF-based HATRIC-LRC without applying the cell surface filter list prior to quantitative data analysis**

|  |
| --- |
| **Supplementary Figure 4:** **Protter image of EGFR.** 19 peptides of EGFR were identified and quantified (**Fig. 2a**) spanning extracellular and intracellular domains of EGFR. Identified peptides are highlighted in blue, whereas known N-glycosylation sites of EGFR are highlighted in green. The image was generated using protter [5]. |

|  |
| --- |
| **Supplementary Figure 5:** **TRICEPS-LRC with anti-EGFR antibody and holo-transferrin on 50 million MDA-MB 231 cells.** We employed the originally published experimental conditions to perform TRICEPS-LRC [6] and successfully identified EGFR (highlighted in blue). |

|  |
| --- |
| **Supplementary Figure 6: TRICEPS-LRC with anti-EGFR antibody and holo-transferrin on 1 million MDA-MB 231 cells.** We used the originally published experimental conditions to perform TRICEPS-LRC [6] and were not able to identify EGFR using anti-EGFR Antibody (highlighted in blue) on 1 million MDA-MB 231 cells. |

| 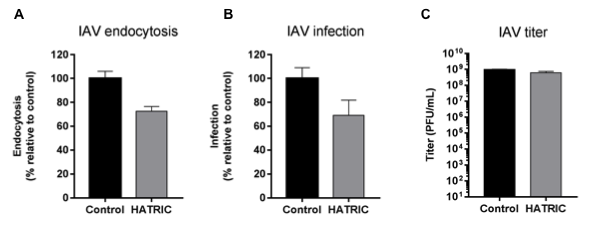 |
| --- |
| **Supplementary Figure 7: Impact of HATRIC-coupling to influenza on efficiency of viral endocytosis.**  A, IAV endocytosis. B, infectivity. C, IAV titer. IAV particles were left unchanged (control) or coupled to HATRIC (HATRIC) and submitted to endocytosis assay (25 min post warming) and infection assay (7 hpi) as described previously [7]. For IAV titration, control and HATRIC-conjugated virus was infected in a 10-fold serial dilution series onto a monolayer of MDCK II cells and overlaid with 1.2 % Avicel containing MEM. Plaques were counted after 3 days of infection and the plaque forming unit (PFU) was calculated per mL of inoculant . |

| 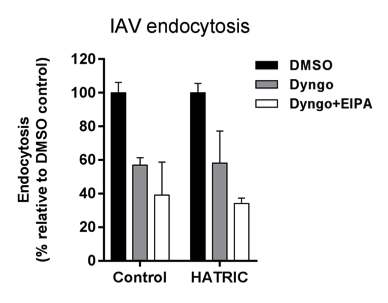 |
| --- |
| **Supplementary Figure 8**: **Results of qPCR from siRNA-transfected cells.** A549 cells were pretreated with Dyngo-4a (50μM) or both Dyngo-4a and EIPA (80 μM) for 30 min, after which equal volumes of IAV were bound for 45 min on ice in the presence of the drug(s). The cells were then washed and incubated at 37°C for 25 min in the presence of the drug(s), fixed and stained for endocytosis analysis [7]. |

| 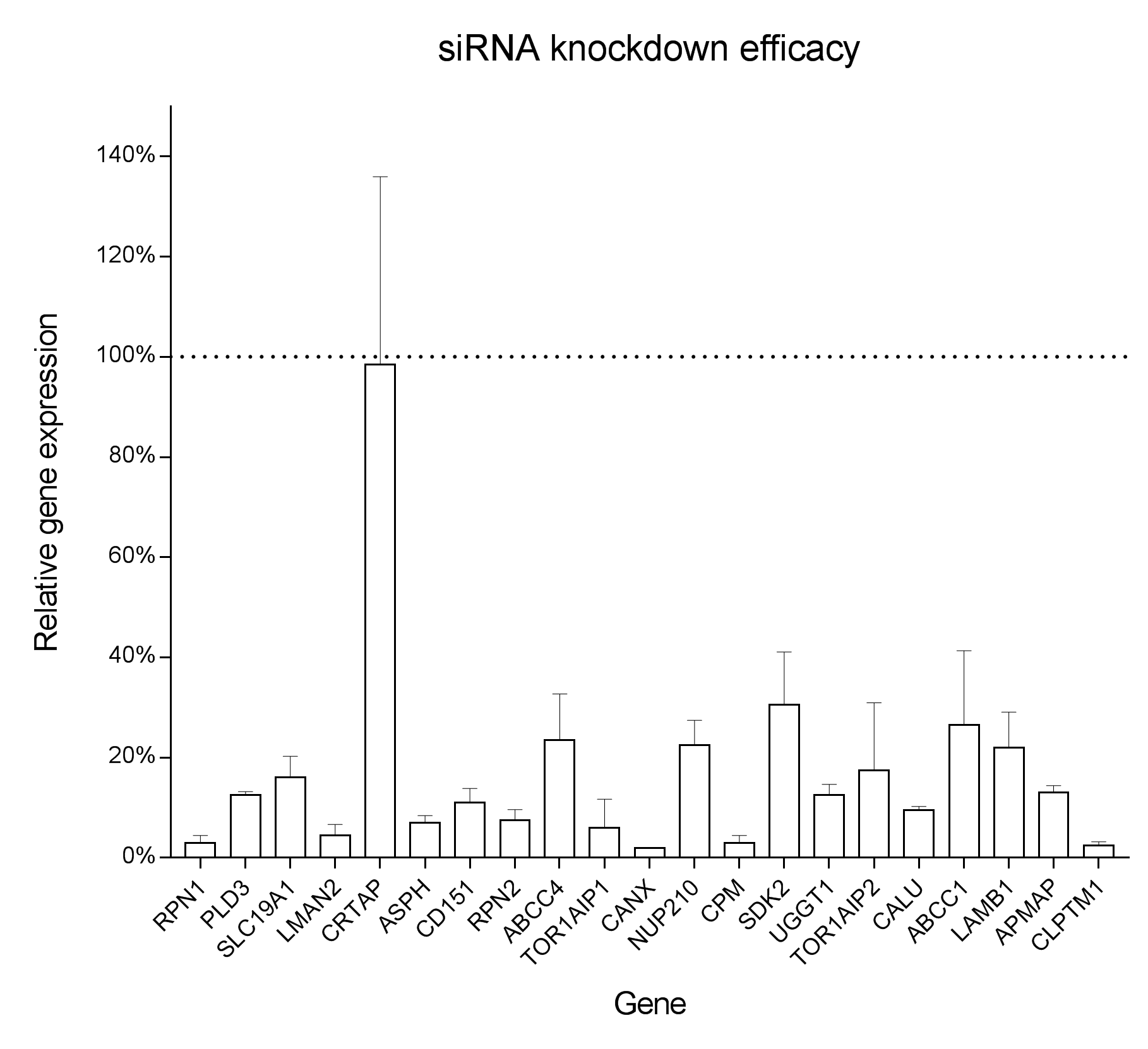 |
| --- |
| **Supplementary Figure 9: Results of qPCR from siRNA-transfected cells.** siRNA-mediated silencing of IAV-interacting candidates was assessed using a ∆∆Ct method to determine the relative gene expression from qPCR data using HPRT as the housekeeping gene. For all genes tested, siRNA-mediated knockdown resulted in 70-98% reduction in mRNA levels compared to non-targeting siRNA control. The bars represent relative gene expression relative to the control taken from biological duplicates with standard deviation. The experiment was repeated twice with similar results. |

Supplementary Table 1: List of potential EGF interaction partner candidates on H-358 cells.

| **Protein Name** | **Gene Name** | **Uniprot Accession** | **Log2-transformed Fold Change (EGF/glycine)** | **FDR-adjusted p-value** | **Length** |
| --- | --- | --- | --- | --- | --- |
| Pro-epidermal growth factor (EGF) | EGF | P01133 | 5.05 | 0.015 | 1207 |
| Transmembrane emp24 domain-containing protein 7 | TMED7 | Q9Y3B3 | 1.87 | 0.016 | 224 |
| Epidermal growth factor receptor | EGFR | P00533 | 1.66 | 0.00005 | 1210 |
| 3-ketoacyl-CoA thiolase, peroxisomal | ACAA1 | P09110 | 1.12 | 0.014 | 424 |
| Monocarboxylate transporter 4 | SLC16A3 | O15427 | 1.05 | 0.003 | 465 |
| Sarcoplasmic/endoplasmic reticulum calcium ATPase 1 (SERCA1) | AT2A1 | O14983 | 0.62 | 0.040 | 1001 |
| Filamin-A | FLNA | P21333 | 0.59 | 0.004 | 2647 |

Supplementary Table 2: List of potential H3N2 interaction partner candidates on A549 cells.

| **Protein Name** | **Gene Name** | **Uniprot Accession** | **log2-transformed fold change (Virus/Insulin)** | **FDR-adjusted p-value** |
| --- | --- | --- | --- | --- |
| Carboxypeptidase M | CPM | P14384 | 3.00 | 0.0007 |
| Calumenin | CALU | O43852 | 1.92 | 0.0007 |
| Nuclear pore membrane glycoprotein 210 | NUP210 | Q8TEM1 | 1.94 | 0.0032 |
| Aspartyl/asparaginyl beta-hydroxylase | ASPH | Q12797 | 1.07 | 0.0032 |
| Vesicular integral-membrane protein VIP36 | LMAN2 | Q12907 | 0.81 | 0.0032 |
| Protein sidekick-2 | SDK2 | Q58EX2 | 5.89 | 0.0035 |
| Multidrug resistance-associated protein 4 | ABCC4 | O15439 | 1.35 | 0.0039 |
| Folate transporter 1 | SLC19A1 | P41440 | 2.61 | 0.0042 |
| Inhibitor of nuclear factor kappa-B kinase-interacting protein | IKIP | Q70UQ0 | 1.51 | 0.0042 |
| CD151 antigen | CD151 | P48509 | 0.78 | 0.0065 |
| Torsin-1A-interacting protein 1 | TOR1AIP1 | Q5JTV8 | 2.02 | 0.0097 |
| Cleft lip and palate transmembrane protein 1 | CLPTM1 | O96005 | 1.99 | 0.0107 |
| Multidrug resistance-associated protein 1 | ABCC1 | P33527 | 1.10 | 0.0110 |
| Cartilage-associated protein | CRTAP | O75718 | 2.62 | 0.0119 |
| Calnexin | CANX | P27824 | 0.80 | 0.0135 |
| UDP-glucose:glycoprotein glucosyltransferase 1 | UGGT1 | Q9NYU2 | 2.40 | 0.0159 |
| Laminin subunit beta-1 | LAMB1 | P07942 | 1.03 | 0.0160 |
| Dolichyl-diphosphooligosaccharide--protein glycosyltransferase subunit 1 | RPN1 | P04843 | 1.39 | 0.0168 |
| Dolichyl-diphosphooligosaccharide--protein glycosyltransferase subunit 2 | RPN2 | P04844 | 1.76 | 0.0171 |
| Torsin-1A-interacting protein 2 | TOR1AIP2 | Q8NFQ8 | 1.97 | 0.0200 |
| Adipocyte plasma membrane-associated protein | APMAP | Q9HDC9 | 0.87 | 0.0208 |
| Equilibrative nucleoside transporter 1 | SLC29A1 | Q99808 | 1.15 | 0.0278 |
| Calreticulin | CALR | P27797 | 1.26 | 0.0305 |
| Phospholipase D3 | PLD3 | Q8IV08 | 1.09 | 0.0326 |

Supplementary References

1. [Liu, W. *et al.* A rapid and fluorogenic TMP-AcBOPDIPY probe for covalent labeling of proteins in live cells. *J. Am. Chem. Soc.* **136,** 4468–4471 (2014).](http://paperpile.com/b/IkSzMB/bRw7)

2. [Teng, B., Bai, Y., Chang, Y., Chen, S. & Li, Z. Technetium-99m-labeling and synthesis of thymidine analogs: potential candidates for tumor imaging. *Bioorg. Med. Chem. Lett.* **17,** 3440–3444 (2007).](http://paperpile.com/b/IkSzMB/qt6v)

3. [Yu, T.-B., Bai, J. Z. & Guan, Z. Cycloaddition-promoted self-assembly of a polymer into well-defined beta sheets and hierarchical nanofibrils. *Angew. Chem. Int. Ed Engl.* **48,** 1097–1101 (2009).](http://paperpile.com/b/IkSzMB/IlKL)

4. [Willibald, J., Harder, J., Sparrer, K., Conzelmann, K.-K. & Carell, T. Click-modified anandamide siRNA enables delivery and gene silencing in neuronal and immune cells. *J. Am. Chem. Soc.* **134,** 12330–12333 (2012).](http://paperpile.com/b/IkSzMB/MfYn)

5. [Omasits, U., Ahrens, C. H., Müller, S. & Wollscheid, B. Protter: interactive protein feature visualization and integration with experimental proteomic data. *Bioinformatics* **30,** 884–886 (2014).](http://paperpile.com/b/IkSzMB/9d1s)

6. [Frei, A. P. *et al.* Direct identification of ligand-receptor interactions on living cells and tissues. *Nat. Biotechnol.* **30,** 997–1001 (2012).](http://paperpile.com/b/IkSzMB/VKDP)

7. [Banerjee, I., Sbalzarini, I. F., Horvath, P. & Helenius, A. Histone deacetylase 8 is required for centrosome cohesion and influenza A virus entry. *PLoS* (2011).](http://paperpile.com/b/IkSzMB/uS1I)
