# Supplementary material for: HATRIC-based identification of receptors for orphan ligands
Source: Nat Commun. 2018 Apr 17;9:1519. doi: 10.1038/s41467-018-03936-z (PMC5904110; doi:10.1038/s41467-018-03936-z)
Supplement: Supplementary file 2 — Description of Additional Supplementary Files [file 41467_2018_3936_MOESM2_ESM.pdf]

Descriptions of Additional Supplementary Files

File Name: Supplementary Data 1
Description: Quantification data for EGF-based HATRIC-LRC on 20 million H-358 cells without applying surfaceome filter

File Name: Supplementary Data 2
Description: Significance testing result for EGF-based HATRIC-LRC on 20 million H-358 cells without applying surfaceome filter

File Name: Supplementary Data 3
Description: List of cell surface proteins for data filtering prior to quantitative analysis in HATRIC-LRC

File Name: Supplementary Data 4
Description: Quantification data EGF-based HATRIC-LRC on 20 million H-358 cells with application of cell surface protein filter

File Name: Supplementary Data 5
Description: Significance testing result for EGF-based HATRIC-LRC on 20 million H-358 cells with application of cell surface protein filter

File Name: Supplementary Data 6
Description: Quantification data for anti-EGFR antibody- and holo-transferrin-based HATRICLRC on 1 million MDA-MB231 cells.

File Name: Supplementary Data 7
Description: Significance testing result for anti-EGFR antibody- and holo-transferrin-based HATRIC-LRC on 1 million MDA-MB231 cells.

File Name: Supplementary Data 8
Description: Quantification data for anti-EGFR antibody- and holo-transferrin-based TRICEPSLRC on 1 million MDA-MB231 cells.

File Name: Supplementary Data 9
Description: Significance testing result for anti-EGFR antibody- and holo-transferrin-based TRICEPS-LRC on 1 million MDA-MB231 cells.

File Name: Supplementary Data 10
Description: Quantification data for folate-based HATRIC-LRC

File Name: Supplementary Data 11
Description: Significance testing result for folate-based HATRIC-LRC.

File Name: Supplementary Data 12
Description: Quantification data for H3N2-based HATRIC-LRC.

File Name: Supplementary Data 13
Description: Significance testing result for H3N2-based HATRIC-LRC.

File Name: Supplementary Data 14
Description: qPCR data from the siRNA-transfected cells (tab 1) and sequences of primers used for the qPCR (tab 2).

File Name: Supplementary Data 15
Description: Results of statistical significance testing for siRNA depletion experiment.
